# Direct Laser Writing of Transparent Polyimide Film for Supercapacitor

**DOI:** 10.3390/nano10122547

**Published:** 2020-12-18

**Authors:** Fei Huang, Guoying Feng, Jiajia Yin, Sikun Zhou, Li Shen, Shutong Wang, Yun Luo

**Affiliations:** 1 College of Electronics and Information Engineering, Sichuan University, Chengdu 610065, China; 2018222050051@stu.scu.edu.cn (F.H.); zhousikun@stu.scu.edu.cn (S.Z.); 2019222050018@stu.scu.edu.cn (L.S.); luoyun809@caep.cn (Y.L.); 2 Institute of Optics and Electronics, Chinese Academy of Sciences, P.O. Box 350, Chengdu 610209, China; yinjj@ioe.ac.cn

**Keywords:** direct laser writing, supercapacitor, transparent polyimide film

## Abstract

Direct laser writing (DLW) is a convenient approach for fabricating graphene-based flexible electronic devices. In this paper, laser-induced graphene was successfully prepared on a thin and transparent polyimide film through the DLW process. Experiments have demonstrated that interdigital thin film capacitor prepared by the DLW method has a high specific capacitance of 8.11 mF/cm^2^ and volume capacitance density of 3.16 F/cm^3^ (0.05 mA/cm^2^) due to the doped fluoride in the laser-induced graphene. The capacitance is about 20 times larger than the super-capacitor based non-transparent polyimide film of the same thickness. Owing to its thin, flexible, higher electrochemical characteristics, the transparent polyimide film is promising for integrating and powering portable and wearable electronics.

## 1. Introduction

Flexible electronics show great promise to enable a variety of new applications for energy conversion and storage, food security tags, environmental monitoring, personalized healthcare, and bioinspired soft robotics [1,2,3,4,5]. The graphene is widely used as an ideal electrode material to develop supercapacitors [6], sensors [7,8,9], transistors [10,11], and photodetectors [12,13], owing to its extremely high specific surface area (2630 m^2^ g^−1^) and excellent conductivity (200 S m^−1^). The materials for fabricating devices and graphene preparation method are of great importance to improve device performance and control process cost.

A number of materials have been reported to fabricate graphene devices, including lignocellulose [14], lignin [15], phenolic resin [16], polydimethylsiloxane (PDMS) [17], polyether ether ketone (PEEK) [18], polysulfone-class polymers [19], polytetrafluoroethylene (PTFE) [20], polyetherimide (PEI) [21], and polyimide film (PI) [22]. These materials are mainly high-temperature engineering plastics and cross-linked polymers which have better performance in converting into LIG (Laser-Induced Graphene) [23]. Under laser irradiation, the polymers can be selectively modified into electrically conductive structures by photo-thermal and/or photo-chemical effects. The general methods for graphene preparation, including graphite oxide reduction [24], liquid-phase exfoliation [25], electrochemical exfoliation [26], chemical vapor deposition (CVD) [27], and epitaxial growth [28], complicate the operational procedures and increase the process costs. Comparing with the conventional techniques, such as inkjet printing, conventional lithographic, layer-by-layer assembly, and screen printing [29,30,31,32], direct laser writing (DLW) technology is much more suitable for high-throughput and large-scale fabrication of inexpensively flexible graphene electronics due to the non-contact and maskless fabrication.

In 2014, Lin et al. [22] used a CO_2_ laser to prepare three-dimensional porous graphene electrodes on a non-transparent PI by DLW. The result showed that the DLW approach has a number of advantages, such as simple operation, fast processing speed, and high patterning accuracy. However, because of the presence of conjugate aromatic ring structure on the main chain, non-transparent PI is easy to form intramolecular and intermolecular charge transfer complex, so the film has a poor light transmission in the visible light region. Fluorine-containing groups can be expected to introduce to avoid or reduce conjugated units in the polyimide structure, which reduce the transmission of loads within or between molecules to increase the transmission. Due to better optical transparency and lower absorption, transparent PIs have been considered for potential applications in aerospace, modern microelectronics, photoelectronics, and wearable devices. To the best of our knowledge, there is no relevant literature about the graphitization of transparent PIs by laser irradiation and corresponding application can be found.

Aimed at investigating the electrochemical characteristics of supercapacitor induced on the transparent PI by a handy method, a heteroatom-doped transparent polyimide film was used in this paper, and the graphene was successfully induced on it using the DLW process. Present work enriches the research of low-cost and high-efficacy fabrication of graphene electrodes for the next generation of flexible electronics. The desirable electrochemical performance and stable cycle capacity prove its efficient capacitance characteristics. Furthermore, the attributes of small size, thin thickness, and flexibility of the device ensures a potential possibility that it can be used to prepare miniaturized, flexible, and wearable supercapacitors.

## 2. Experiment

### 2.1. Preparation and Characterizations

The transparent PI preparation process began from the resin synthesis using the polyaddtion of equimolar 2,2′-bis(trifluoromenthyl)-[1,1′-biphenyl]-4,4′-diamine (TFDB) and 3,3′,4,4′-Biphenyl tetracarboxylic dianhydride (BPDA). The TFDB was dissolve in dimethylacetamide (DMAc) at room temperature. Then, dianhydride BPDA was added to the solution with continuous stirring. The reaction mixture was stirred for 30 min at low temperature and then left to react overnight at room temperature. The concentration of the solution would be controlled in 12% (wt). Finally, the transparent PI was prepared by thermal imidization. The non-transparent PI was prepared using polyvinyl alcohol (PVA) and thermal imidization.

**Figure 1:**
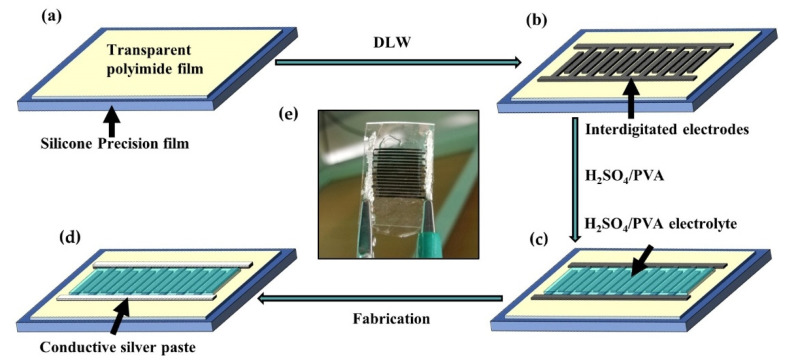
Schematic illustration of fabrication process of (**a**–**d**) supercapacitor and (**e**) is physical image of supercapacitor.

The porous carbon materials prepared by DLW on transparent PI and non-transparent PI are denoted as DLWT and DLWP, respectively. Base on them, the planar interdigital supercapacitors were further fabricated. The fabrication process displayed in Figure 1a–d includes the following four steps: (i) The transparent PI was smoothly covered on silicone precision film (BD Film KYN-500) substrate, and then it was wiped carefully. (ii) A 450 nm semiconductor laser was used to engrave the interdigitated electrodes pattern on the transparent PI with an engraving speed of 100 mm/s under 1750 mW. Each side of the interdigital electrode was composed of 8 pairs of interdigital microelectrodes, and the total area of the interdigital region is 2.625 cm^2^. (iii) The H_2_SO_4_/PVA (polyvinyl alcohol) electrolyte was added onto the active areas, to ensure that the electrolyte diffuses into the electrodes through drying overnight. (iv) Conductive silver paste was used to make good contact with the carbon electrodes. The H_2_SO_4_/PVA hydrogel electrolyte was prepared similarly to a reported procedure [33]. Finally, the supercapacitor with the same process was made based on the non-transparent PI film for comparison. Simply speaking, the difference between transparent and non-transparent films can be attributed to that transparent film contains fluorine, while non-transparent film does not contain fluorine. The prepared supercapacitor is displayed in Figure 1e.

The DLWT and DLWP were further characterized by Raman spectroscopy (LabRAM HR, HORIBA, Paris, France), scanning electron microscope (SEM) (Quanta 650FEG, FEI, Hillsboro, OR, USA), electron diffraction spectroscopy (EDS) (Quanta 650FEG, FEI, Hillsboro, OR, USA), and X-ray photoelectron spectroscopy (XPS) (Escalab 250Xi, Thermo Scientific, Waltham, MA, USA). The DLWT was observed by a SEM in high vacuum mode with a 10 kV accelerating voltage and 500–30,000 times magnification. EDS was tested to analyze the types and contents of DLWT and DLWP. Raman spectra was obtained on a Raman spectrometer with an excitation laser at 532 nm. The XPS analysis was carried out using Al-Kα target (ħ*ν* =1486.6 eV) under 150 W power and 350 keps sensitivity. The vacuum degree of the analysis chamber was 10^−8^ mbar, the angle between the detector and the sample surface was 90°, and the analysis area was 700 × 300 μm.

### 2.2. Electrochemical Measurements

The electrochemical capability of the DLWT and DLWP was tested by employing a cyclic voltammetry (CV) test, a galvanostatic charge/discharge (GCD) test, and an electrochemical impedance spectroscopy (EIS) test. They were implemented on an electrochemical workstation (660E, CHI, Columbus, OH, USA). Both CV and GCD tests were equipping with a two-electrode setup. The frequency range of EIS test was conducted from 0.01 Hz to 100 kHz, and the amplitude was 10 mV at the open circuit potential.

The areal specific capacitances (*C_A_*, mF/cm^2^) of supercapacitors calculated from CV curves are based on the following Equation [22]:

(1) $$C_{A}=\frac{1}{2\times S\times v\times (V_{f}-V_{i})}\int_{V_{i}}^{V_{f}}I(V)dV,$$

where *S* is the area of the active electrodes (in cm^2^), *v* is the voltage scan rate (in V s^−1^), *V*_f_ and *V*_i_ are the potential limits of the CV curves, and *I*(*V*) is current at different potentials. $\int_{V_{i}}^{V_{f}}I(V)dV$ is the numerically integrated area of the CV curves.

The areal specific capacitances (*C_A_*, mF/cm^2^) of supercapacitors calculated from GCD curves are based on the following Equation [22]:

(2) $$C_{A}=\frac{I}{S\times (dV/dt)},$$

(3) $$C_{V}=\frac{C_{A}}{d},$$

where *I* is the discharge current (in amperes), *S* is the area of the active electrodes, *dV*/*dt* is the slope of galvanostatic discharge curves. *C_V_* is the volume specific capacitances (mF/cm^2^), and *d* is the thickness of active materials.

The specific power density (*E_A_*, in Wh/cm^2^) and the specific energy density (*P_A_*, in W/cm^2^) were obtained from Equations (4) and (5) [22]:

(4) $$E_{A}=\frac{1}{2}\times C_{A}\times \frac{{\left(\Delta V\right)}^{2}}{3600},$$

(5) $$P_{A}=3600\times \frac{E_{A}}{\Delta t},$$

where ∆*V* is the discharge potential range, and ∆*t* is the discharge time.

## 3. Results and Discussions

### 3.1. Characteristics of Materials

**Figure 2:**
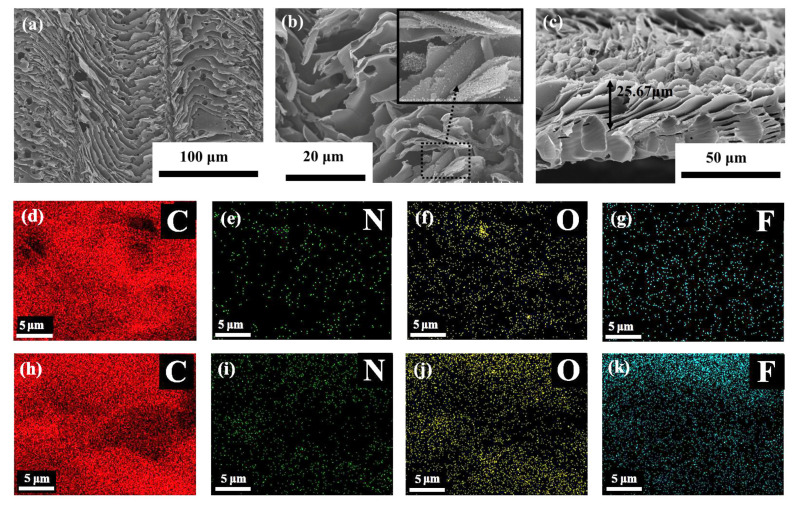
(**a**–**c**) The scanning electron microscope (SEM) images of the porous carbon materials prepared by DLW on transparent PI (DLWT)’s; (**a**–**b**) surface and (**c**) thickness cross-section; (**d**–**g**) the electron diffraction spectroscopy (EDS) mapping of C, N, O, F of DLWT surface; (**h**–**k**) the EDS mapping of C, N, O, F of thickness cross-section.

As shown in Figure 2a, the laser scanning paths on the surface of DLWT appear as horizontal stripes, and the adjacent stripes are closely arranged. Under laser irradiation, the PI transform to LIG goes a photothermal process which is associated with the localized high temperature and pressure produced, which causes the pyrolysis of materials. The pyrolysis leads to a rapid release of gas, which causes the carbonized structure to appear as a compact sheet-like stacked and porous structures. The center of the laser beam has a high heat source density, and the two sides have a low heat source density, resulting in higher carbonization degree in the stripes’ center than in the two sides. Thus, a certain groove structures can be observed between adjacent carbonized stripes. Figure 2b is a compact sheet-like stacked structure of DLWT. The porous structure on the sheet-like surface is formed by the rapid release of gas by-product. Figure 2c is the cross section of DLWT and the thickness of the carbonization is about 25.67 μm. Figure 2d–g shows the EDS mapping of DLWT surface, and the C, N, O, and F contents are 96%, 1%, 2%, and 1%, respectively. Compared with the thickness section (Figure 2h–k), the contents of each element are 86%, 2%, 5%, and 8%, respectively.

**Figure 3:**
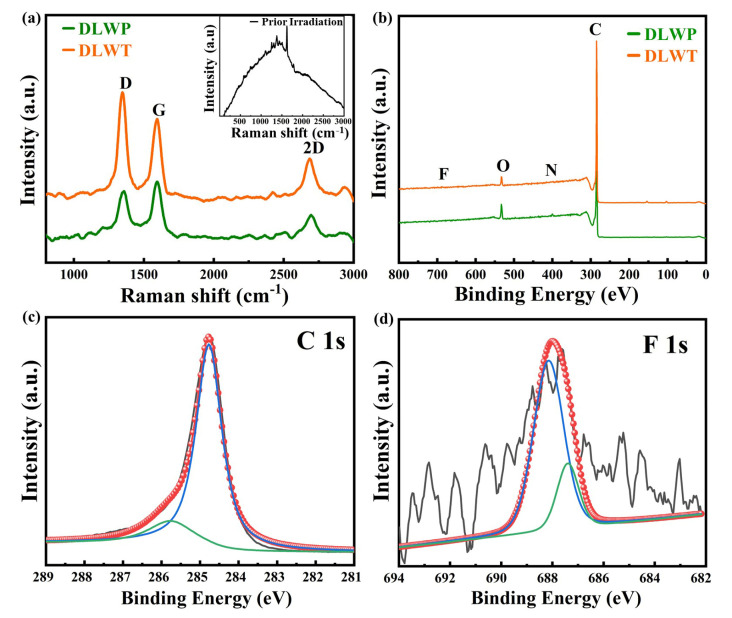
(**a**) the Raman spectra of the porous carbon materials prepared by DLW on transparent PI and non-transparent PI (DLWT and DLWP) (Embedded graph is the Raman spectra of transparent polyimide film (PI)); (**b**) the X-ray photoelectron spectroscopy (XPS) spectra; (**c**) the high-resolution C 1s of DLWT; (**d**) the high-resolution F 1s of DLWT.

The Raman spectrum of the DLWT and DLWP are shown in Figure 3a (with embedded graph indicating the prior irradiation spectra of transparent PI), the prior irradiation spectra of non-transparent PI can be referred to some previous reports [34,35]. After carbonization of graphene materials induced by laser, the imide group of PI transformed to the six-membered ring structure. Figure 3a exhibits the characteristics of graphene after carbonization, and three prominent peaks can be observed: (i) the D peak at 1350 cm^−1^, (ii) the G peak at 1590 cm^−1^, and (iii) the 2D peak at 2700 cm^−1^. The G peak is the main characteristic peak of graphene, which is caused by the in-plane vibration of sp^2^ carbon atoms. This peak can effectively reflect the number of graphene layers, but it is extremely susceptible to stress. The D peak is generally considered to be the disordered vibration peak of graphene. The specific position of the peak is related to the laser wavelength. It is caused by the lattice vibration leaving the center of the Brillouin zone and is used to describe structural defects or the edge in the graphene sample. The 2D peak is the second-order Raman peak of two-phonon resonance, which is used to describe the interlayer stacking of carbon atoms in the graphene sample. The peak frequency is also affected by the laser wavelength [36]. Since the defect density is proportional to *I*_D_/*I*_G_, in a high defect density regime graphene structure, *I*_D_ will decrease with respect to *I*_G_. The Raman spectra shows the *I*_D_/*I*_G_ of DLWT and DLWP are 1.32 and 0.92, respectively, which implies that the DLWP contains more defect than DLWT [37,38].

Figure 3b–d exhibit the full survey X-ray photoelectron spectroscopy (XPS) spectrum on the surface of DLWT and DLWP, revealing the presence of C, N, O, and C, N, O, F, that is consistent with the EDS mapping. The F and N element signals are somewhat non-obvious in the XPS spectrum of DLWT. We infer that the N may be displaced by the introduction of F during the laser carbonization process. In addition, some F and N element may volatilize as a gas during pyrolysis, resulting in a content decline. From the DLWP’s XPS spectrum, as shown in Figure 3c, the high-resolution C 1s spectrum at 285.74 eV has a C–F bond. Similarly, the high-resolution spectrum of the F 1s (Figure 3d) can be deconvoluted into two prominent peaks at the binding energy of 687.40 and 688.15 eV, which are all assigned to C–F bond correspondingly.

### 3.2. Electrochemical Performance of the Supercapacitors

**Figure 4:**
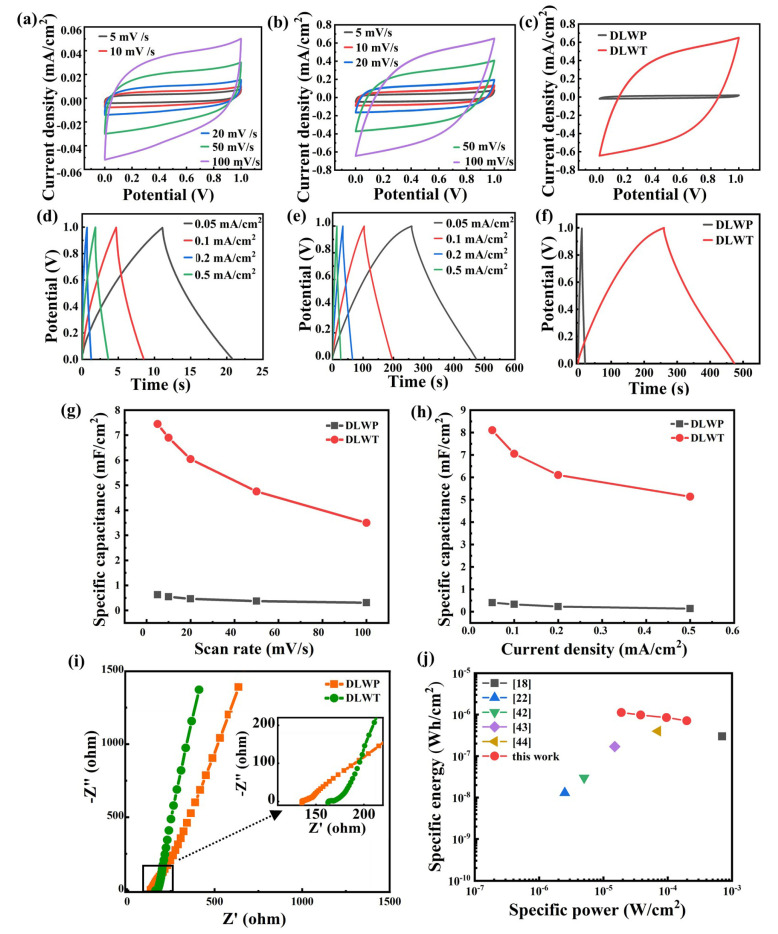
The cyclic voltammetry (CV) curve of (**a**) DLWP and (**b**) DLWT with the scan rates of 5, 10, 20, 50, and 100 mV/s; (**c**) the CV curve comparison between DLWT and DLWP (scan rates is 100 mV/s); The galvanostatic charge/discharge (GCD) curve of (**d**) DLWP and (**e**) DLWT with the current density of 0.05, 0.1, 0.2, and 0.5 mA/cm^2^; (**f**) the GCD curve comparison of DLWT and DLWP (current density is 0.05 mA/cm^2^); specific capacitance (**g**) calculated from CV data (**h**) calculated from GCD data; (**i**) the Nyquist plots of DLWP and DLWT; (**j**) Ragone plot.

Figure 4a,b show the CV curves of DLWP and DLWT at different scan rates, which exhibit the quasi-rectangular feature of capacitance behavior. The CV curve changes from approximately “rectangular shape” to “fish-shape” with the increase of scanning rates, which indicates a good charge and discharge properties at the low scanning rate. Because of the existence of the ohm voltage drop of the laser-carbonized porous electrode, the electrode needs a certain period of time to stabilize, forming a rounded corner in the CV curves. Figure 4c is the CV curve comparison between DLWT and DLWP with scan rates of 100 mV/s. The GCD curves of DLWP and DLWT of various current densities illustrated in Figure 4d,e are almost triangular, indicating double-layer-like capacitance behavior. Figure 4f shows the GCD curve comparison between DLWT and DLWP with current density of 0.05 mA/cm^2^. In Figure 4g, the DLWP’s specific capacitance calculated by the CV curve are 0.629, 0.547, 0.468, 0.3758, and 0.3099 mF/cm^2^, with the scan rates increasing from 5 to 100 mV/s, and of the value for DLWT are 7.45, 6.89, 6.05, 4.755, and 3.50 mF/cm^2^, respectively. The specific capacitance of DLWT is about 12 times higher than DLWP based on the CV curve. Figure 4h shows the specific capacitance calculated by the GCD curve, where the current density increases from 0.05 to 0.5 mA/cm^2^, the values for DLWP are 0.4066, 0.3307, 0.232, 0.141 mF/cm^2^, and for DLWT are 8.11, 7.05, 6.11, 5.14 mF/cm^2^, respectively. The specific capacitance of DLWT is 20 times higher than that of the DLWP based on the GCD curves. The specific capacitance is also higher than recent supercapacitors related studies based on the DLW method [16,34,39]. The carbonization depth of DLWT is only 25.67 μm, so the calculated bulk capacitance of DLWT and GCD curve are 2.9, 2.68, 2.36, 1.85, and 1.36 F/cm^3^ from CV curve and are 3.16, 2.75, 2.38, and 2.00 F/cm^3^ from GCD curve, which is comparable to the values of several recently reported graphene-based super-capacitors [18,40,41].

Figure 4i shows the Nyquist curves of DLWP and DLWT. The figure illustrates that the slope of the Nyquist curve of the DLWT is higher than that of the DLWT, which means the DLWT has lower ionic resistance than DLWP. Intercept of Nyquist curve in the horizontal axis (high frequency region) indicates the intrinsic resistance of the electrode. From the figure, DLWT displays a higher ESR (Equivalent Series Resistance) (163.1 Ω) compared with the DLWP (135.7 Ω), which can be presented from a “fish shape” in the CV plots under high scan speed. However, the DLWT still show higher capacitance compared to the DLWP, implying that the fluoride doped LIG may play a role on improving capacitive performance due to the electronegativity of fluoride heteroatom. The Ragone plot (Figure 4j) shows a specific power and energy of supercapacitor preparation from transparent PI and other non-transparent materials, which shows good capacitors performance of this supercapacitor compared with previous materials [18,22,42,43,44]. These results show that supercapacitors made of transparent polyimide film have good capacitance characteristics.

**Figure 5:**
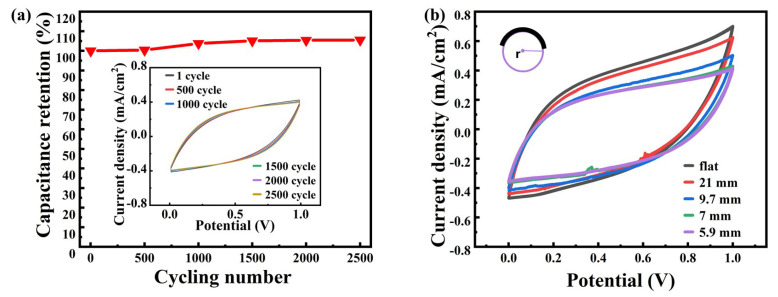
(**a**) Normalized capacitance plot with 2500 charge/discharge cycles (Embedded graph is the initial CV curve after 500, 1000, 1500, 2000, and 2500 cycles number of DLWT with the scan rate of 0.1 V/s.); (**b**) DLWT CV curves of various bending radius under 0.1 V/s scan rate.

The embedded graph in Figure 5a is the CV curve of DLWT which has good cycle stability. As exhibited in Figure 5a, the device maintains 105.5% of its initial capacitance after 2500 cycles. In Figure 5b, when the DLWT is bent, its capacitance performance will decrease slightly. Compared with other published results, DLWT based on transparent polyimide film has favorable electrochemical performance compared with materials made by DLW process on ordinary polyimide film [22]. The above testing results are attributed to the high electronegativity of fluoride, which combines with carbon to form a C–F bond with high polarity and strong stability, thereby improving the overall electrochemistry characters of the LIG material.

## 4. Conclusions

In summary, transparent polyimide film has been successfully carbonized by semiconductor laser. It proved that direct laser writing is an efficient method to induce porous graphene on thin and flexible polyimide film. Moreover, the supercapacitors based on DLWT exhibits a higher electrochemical characteristic than the DLWP, due to the hierarchical porous structures and fluorine doped. The DLWT shows higher specific capacitance that is about 12 times and 20 times larger than the DLWP, respectively, based on CV curve and GCD curve. The facile fabrication and superior performance of carbon-based supercapacitor provides a new avenue for designing of miniaturized and flexible wearable electronic devices.
